# 24435 Pathogen-specific metabolic pathways and innate immune responses associated with Chlamydia trachomatis infection and other STIs

**DOI:** 10.1017/cts.2021.627

**Published:** 2021-03-31

**Authors:** John D. Ryan, Evelyn Toh, Julie A. Brothwell, Yuan Sun, Stephen J. Jordan, David E. Nelson

**Affiliations:** Indiana University School of Medicine

## Abstract

ABSTRACT IMPACT: This project seeks to identify unique host responses that are biomarkers for specific urethral pathogens, and which can be used in the development of point-of-care (POC) STI diagnostics. OBJECTIVES/GOALS: How Chlamydia trachomatis (CT) and other common STIs, e.g. Neisseria gonorrhoeae, evade immunity and elicit pathology in the male urethra is poorly understood. Our objective is to determine how STI-infected urethral epithelial cells, as well as the uninfected ‘bystander’ cells with which infected cells communicate, respond to CT and other STIs. METHODS/STUDY POPULATION: We evaluated how immortalized urethral cell lines - including transduced human urethral epithelial cells (THUECs) - respond to increasing doses of CT infectious particles using in vitro one-step progeny assays performed in the presence or absence of cycloheximide, a drug that inhibits eukaryotic protein synthesis. We will perform concurrent single-cell RNA sequencing (scRNA-seq) and multiplex cytokine analyses to determine how different CT doses impact the transcriptomes of infected and bystander urethral epithelial cells and modulate cytokine production of the overall monolayer. Results of these experiments will inform the feasibility of performing similar analyses in situ using urethral swabs from men with clinically diagnosed urethritis. RESULTS/ANTICIPATED RESULTS: Our results demonstrate that immune-competent urethral cell monolayers strongly resist CT infection, unless most of the cells are simultaneously infected. This suggests that uninfected bystander cells sense CT-infected cells and secrete soluble factors that may act to limit CT proliferation in infected cells and to inform remaining uninfected cells that a potential pathogen is present. We anticipate that our scRNA-seq and cytokine analyses will identify both specific effector pathways that protect against CT and intracellular signals that modulate them. We speculate that these pathways and signals may differ during infection with CT and other STIs. Importantly, we anticipate that our in vitro model of CT infection will be highly representative of in situ immune responses observed in urethras of infected men. DISCUSSION/SIGNIFICANCE OF FINDINGS: In men, common STIs including CT are usually managed syndromically due to a lack of POC diagnostics. By determining how STIs elicit urethral inflammation and identifying countermeasures that STIs use to evade urethral immunity, we can identify host responses that serve as biomarkers for urethritis, generally, and for specific urethral pathogens.

